# Microwave-Enhanced Catalytic Performance of Benzene Oxidation on MOF-Derived Mn/Ce-Co Oxides

**DOI:** 10.3390/molecules30163388

**Published:** 2025-08-15

**Authors:** Shefeng Li, Pengyi Zhao, Ziyang Liu, Chang Wang, Linling Wang, Siyu Ding

**Affiliations:** 1 School of Chemical and Environmental Engineering, Wuhan Polytechnic University, Wuhan 430023, China; lishefeng@163.com (S.L.); zhaopengyiwhpu@163.com (P.Z.); 20230312012@whpu.edu.cn (Z.L.); ccwang0713@163.com (C.W.); 2 Pilot Plant of Eco-Environment Chemical Industry and Carbon-Neutral Transformative Technologies, Wuhan 430023, China; 3 Hubei Provincial Engineering Research Center of Soil and Groundwater Pollution Prevention and Control, Wuhan 430023, China; 4 School of Environmental Science and Engineering, Huazhong University of Science and Technology, Wuhan 430074, China; wanglinling@mail.hust.edu.cn

**Keywords:** benzene oxidation, microwave irradiation, bimetallic MOF, Mn-Co spinel oxides

## Abstract

Microwave-assisted processing has shown tremendous promise in accelerating chemical reactions and reducing energy consumption through targeted dielectric heating. This study develops MOF-derived Mn-Co and Ce-Co oxide catalysts for energy-efficient benzene oxidation via microwave catalysis. The MnCo spinel oxides (particularly MnCo11-400) exhibit superior microwave absorption and catalytic activity due to enhanced oxygen mobility and tailored dielectric properties. Microwave irradiation enables rapid benzene mineralization over the MnCo11-400 catalyst, achieving 78% conversion at 30 W and complete conversion at 50 W, demonstrating exceptional energy efficiency at low power inputs. Microwaves significantly lower the reaction temperature compared to conventional thermal catalysis (ΔT = 100–250 °C). Stability tests confirm robustness over repeated power cycling (80% conversion retained after 3 × 1 h on/off cycles). Furthermore, an adsorption–microwave oxidation synergistic strategy is demonstrated: pre-adsorbed low-concentration benzene (1.15 mmol) at ambient temperature undergoes complete mineralization within 20 min under 30 W microwave irradiation. The intermittent microwave operation achieves equivalent benzene removal to continuous thermal processing while significantly reducing energy demand. This work establishes MOF-derived spinel oxides as high-performance microwave catalysts for low-temperature VOC abatement.

## 1. Introduction

In recent years, global air pollution has become increasingly severe, and the emission of volatile organic compounds (VOCs) has seriously affected the ambient air quality and human health. Studies have shown that [1,2] VOCs are the main precursors of secondary organic aerosols and fine particulate matter, which are important contributors to atmospheric photochemical smog and acid deposition. Under ultraviolet irradiation, VOCs will react with NO_x_ to generate secondary pollutants like ozone, resulting in photochemical pollution [3]. Hexane, heptane, and octane can affect the human central nervous system [4]. Polycyclic aromatic hydrocarbons and many chlorine-containing organic compounds such as benzene, toluene, xylene, styrene, dichlorotoluene, etc., are carcinogenic, teratogenic, and mutagenic [5]. With the increasing environmental issues, the end treatment of VOCs is imperative.

Adsorption [6], condensation [7], catalytic oxidation [8], photocatalytic degradation [9], and biological methods [10] have been applied to control VOC emissions. Among the techniques, the catalytic oxidation method is considered to be one of the most suitable and broad application prospects for treating VOCs with a large concentration variation range due to the low light-off temperature, high conversion and mineralization rate, less secondary pollution, and wide application range.

As one of the energy resources with a high thermal effect and strong dipole polarization ability, microwaves have been widely used in the treatment of solid, liquid, and gas waste in recent years. Microwave energy has been considered an excellent alternative heating source to promote chemical reactions because of its ability to provide fast, selective, and highly efficient volumetric heating to materials [11,12,13,14]. The thermal effect is the main factor of reaction promotion via microwave irradiation [15,16,17,18]. The temperature of microscale “hot spots” formed from microwaves can reach as high as 1200 °C, and the temperature difference can be higher than 200 °C [16]. J. Dobosz et al. [19] used α-Fe_2_O_3_ as the active component of the catalyst, and propane removal efficiency reached 99.9% through the microwave-catalytic method. A microwave single-mode cavity was introduced into the catalytic reactions by Nigar et al. [20], resulting in 99% degradation efficiency of n-hexane. Our previous work [21,22,23] has proven that the microwave-assisted catalytic removal technique is more effective for small-scale VOC sources with low concentrations from the standpoint of energy efficiency. Thus, it will be an innovative reform to apply the microwave technique in VOCs removal, offering significant improvements over conventional heating, including shorter reaction times, greater energy efficiency, and higher yields of products.

The development of microwave catalysts is the key to realizing the microwave-assisted catalytic process. Transition metal oxides have high activity in catalyzing VOC reactions due to their abundant oxygen vacancies, electron transfer, and valence state changes, and they are considered to be potential alternative materials to precious metal catalysts [24]. In addition, previous studies [25,26] have shown that transition metal oxide materials have high dielectric loss, and their microwave heating performance is also quite excellent. On the other hand, metal organic frameworks (MOFs) are composed of metal nodes and organic connectors and have attracted widespread attention due to their high specific surface area, adjustable porosity, diverse structures, and adjustable functional sites, which make MOFs suitable candidates for catalytic materials or precursors [27,28,29,30]. MOF-derived porous metal oxides have highly porous structures and high surface areas, which are conducive to the adsorption of VOCs and reactivity of oxygen species [31,32]. There are few reports on the effective regulation of microwave heating characteristics and catalytic performance by adjusting the metal elements in MOFs.

In this work, to improve the efficiency of the complete catalytic benzene oxidation, we investigated the effect of the composition of bimetallic MOF-derived Ce-Co and Mn-Co oxides and their catalytic properties. The temperature rise characteristics and benzene oxidation activity of the catalysts under microwave irradiation were studied in detail. The effectiveness of the adsorption–oxidation process for complete removal of gaseous benzene at low concentrations, which consists of adsorption and catalytic oxidation via rapid heating, a characteristic of microwave heating, was also investigated.

## 2. Results and Discussion

### 2.1. Structural and Morphology Characterization

**Figure 1:**
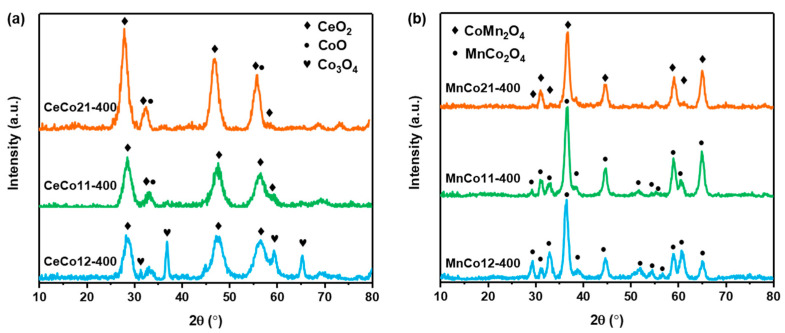
XRD patterns of the (**a**) Ce-Co and (**b**) Mn-Co oxides.

The phase composition of the catalyst was characterized by X-ray diffraction (XRD) patterns in Figure 1. According to the ICDD standard card, the CeCo-300 catalysts were mainly composed of an amorphous phase that could not be identified via XRD measurement (Figure S1). The increase in temperature enhances the crystallinity of the Ce-Co oxide samples. When calcined at 400 °C, the phase composition mainly evolved into CeO_2_(PDF#75-0076)-Co_3_O_4_(PDF#42-1467) for CeCo12-400, CeO_2_-CoO(PDF#75-0419) for CeCo11-400, and CeO_2_-CoO for CeCo21-400 [33]. For MnCo-400 catalysts, the phase composition differs from CeCo-400, with spinel oxide MnCo_2_O_4_(PDF#84-0482) forming in MnCo12-400 and MnCo11-400, while CoMn_2_O_4_(PDF#77-0471) is generated in MnCo21-400 [34,35]. Co-Mn spinel oxide is an excellent oxidation catalyst with higher redox properties, thermal and catalytic stability, and resistance to coke formation than the single oxides [21,22].

**Figure 2:**
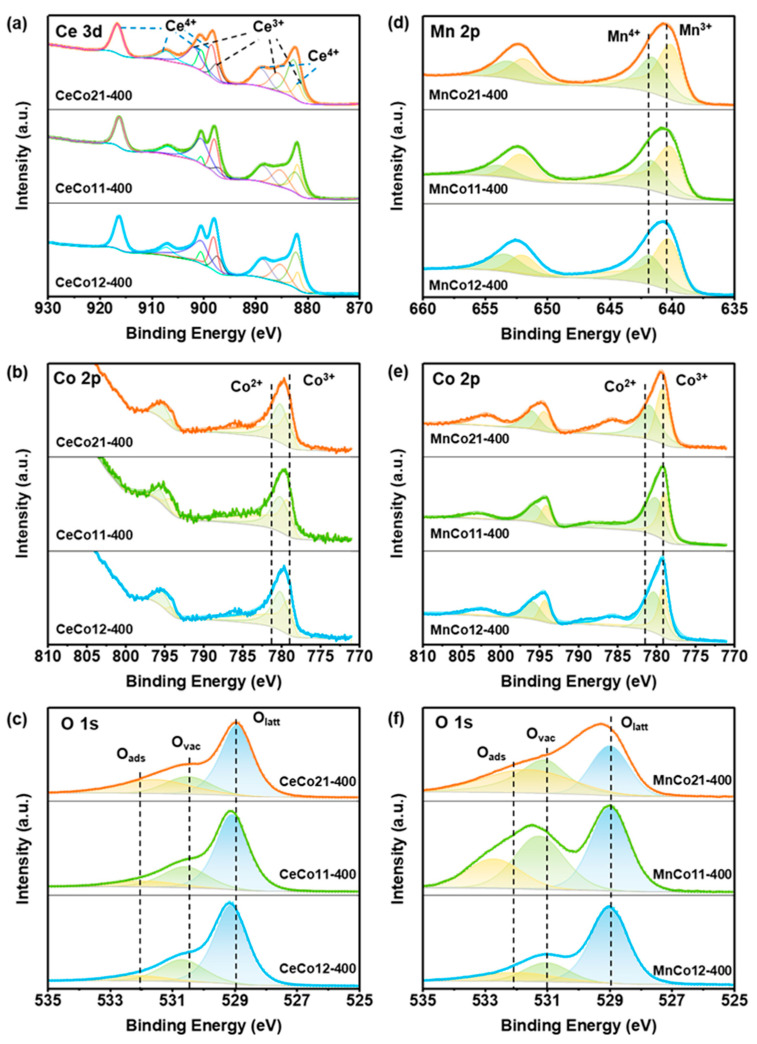
(**a**) Ce 3d, (**b**) Co 2p, and (**c**) O 1s XPS spectra of CeCo12-400, CeCo11-400, and CeCo21-400, (**d**) Mn 2p, (**e**) Co 2p, and (**f**) O 1s XPS spectra of MnCo12-400, MnCo11-400, and MnCo21-400.

Figure 2 and Figure S1 show the XPS spectra of the Ce-Co and Mn-Co oxides. For the CeCo-400, the Ce 3d spectra (Figure 2a) comprise ten peaks corresponding to five pairs of spin-orbit doublets. The peaks located at around 897.8, 881.9, 902.4, and 885.6 eV can be assigned to the v, u, v′ and u′ shake-down final states of Ce^3+^, while the other peaks centered at around 882.8, 901.1, 888.6, 907.2, 898.5, and 916.6 eV can be classified as the v, u, v″, u″, and v″′, u″′ final states of Ce^4+^ [36]. The curves are fitted with Co^2+^ and Co^3+^ components via Co 2p spectra (Figure 2b), characterized by the binding energy located at about 780.8, 796.1 eV and 779.3, 794.4 eV, respectively. The peak 786.0 eV corresponds to the satellite peaks of Co^2+^ species [36]. Peaks with binding energies at 529.0, 531.2, and 532.3 eV in the O 1s spectra (Figure 2c) can be attributed to surface lattice oxygen (O_latt_), oxygen vacancy (O_vac_), and surface adsorption oxygen species (O_ads_), respectively [37]. For the MnCo-400, the peaks in Mn 2p spectra (Figure 2d) around 652.6 eV and 640.7 eV are assigned to Mn 2p_1/2_ and Mn 2p_3/2_ of Mn^3+^ species, respectively, while the peaks around 653.5 eV and 642.1 are assigned to those of Mn^4+^ species [23]. Similar to CeCo-400 samples, the Co^2+^ and Co^3+^ are also indicated in Co 2p (Figure 2e), O_latt_, O_vac_, and O_ads_ are indicated in O 1s spectra (Figure 2f). Moreover, the results calculated based on XPS spectra are summarized in Table 1. The molar ratios of Mn^3+^/Mn^4+^ followed the order of MnCo11-400 (1.20) > MnCo12-400 (1.17) > MnCo21-400 (0.91). A high ratio of Mn^3+^ in catalysts weakens Mn-O bonds on the surface, facilitating oxygen release for oxidation reactions and enhancing the dissociation and activation of surrounding oxygen atoms. The proportion of Mn^3+^ is also in agreement with the concentration of surface oxygen vacancies (O_vac_%), which increase to maintain electrostatic balance as the Mn^3+^ content rises. Consequently, the catalytic properties are governed by the degree of charge-transfer imbalance, the relative concentration of Mn^3+^/Mn^4+^ redox couples, and the oxygen vacancy density. The Ce^3+^/Ce^4+^ and O_vac_% of Ce-Co oxides are lower than that of Mn-Co oxides, which may be one of the reasons for the lower catalytic performance.

**Figure 3:**
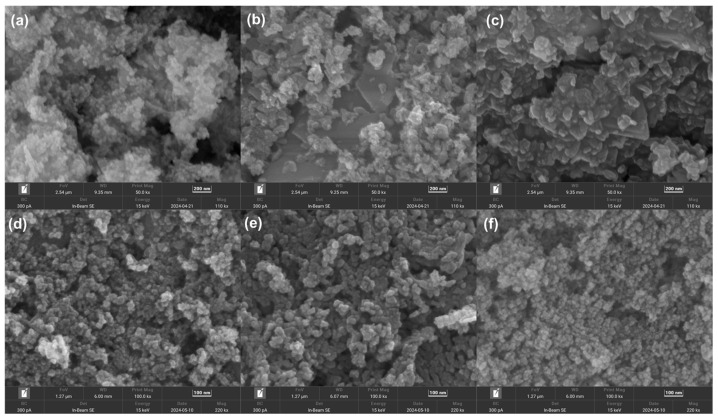
SEM images of the as-prepared samples: (**a**) CeCo12-400, (**b**) CeCo11-400, (**c**) CeCo21-400, (**d**) MnCo12-400, (**e**) MnCo11-400, and (**f**) MnCo21-400.

**Figure 4:**
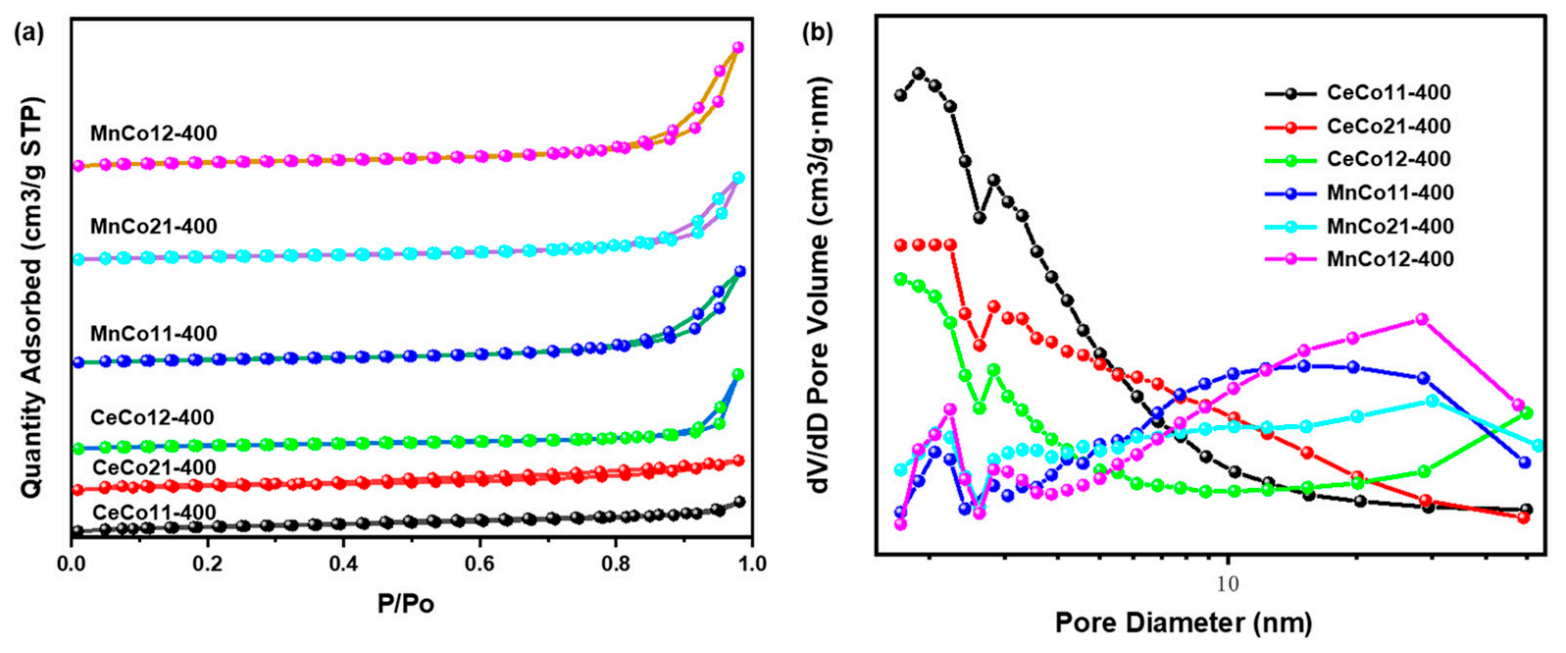
(**a**) Nitrogen adsorption–desorption isotherms and (**b**) pore size distribution of the CeCo-400 and MnCo-400.

Figure 3 shows the surface morphologies of the Ce-Co, Mn-Co oxides calcined at 400 °C. For the Ce-Co oxides calcined at 300 °C (Figure S3), they crystallized into regular-shaped truncated rhombohedral prisms with a different size. The mean length along the long edge of CeCo12-300 is about 1 μm, exceeding those of CeCo11-300 (≈0.5 μm) and CeCo21-300 (≈0.2 μm). Exposure to 400 °C during calcination led to a significant transformation in the morphology of CeCo-400 and MnCo-400, resulting in the partial collapse of the initial rhombic structure and the subsequent development of porous aggregated nanoparticles. The calcination-induced transformation from initial rhombic structures to porous aggregated nanoparticles is beneficial to the enhanced catalytic performance for benzene oxidation, which was in agreement with the experimental results (Figure 4a and Figure S3). On the one hand, the hierarchical pore network facilitates efficient reactant diffusion and accessibility to active sites [38]. Nanoparticle interfacial boundaries create defect-rich zones with a higher oxygen vacancy concentration, promoting oxygen spillover at the interfaces of the catalyst for accelerated redox cycling [39]. Moreover, the aggregated architecture provides exceptional thermal stability while optimizing microwave coupling for energy-efficient oxidation [21,40]. The N_2_ adsorption–desorption isotherm and the pore size distribution of CeCo-400 and MnCo-400 catalysts are plotted in Figure 4. The N_2_ adsorption–desorption isotherms of the catalysts exhibit a combination of Type I and IV characteristics, indicative of a hierarchical microporous/mesoporous structure. And the presence of mesopores is confirmed through the observation of hysteresis loops in the adsorption/desorption branches and the absence of an adsorption plateau at high relative pressures. Mesopores primarily originate from interparticle voids [41,42]. The specific BET surface area (S_BET_) and pore data are presented in Table 2. The S_BET_ of CeCo-400 is slightly higher than that of MnCo-400, while larger mesopores were observed in MnCo-400 (20~22 nm) with a larger pore volume.

### 2.2. Catalytic Performance of Benzene Oxidation

**Figure 5:**
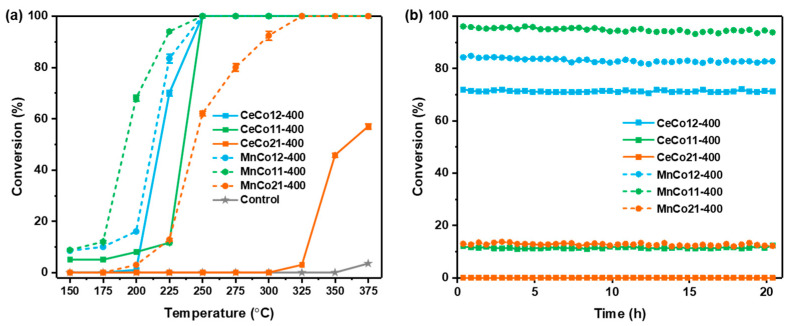
Catalytic activity, (**a**) benzene conversion at different temperatures, and (**b**) reaction stability at 225 °C for 20 h of Ce-Co, Mn-Co oxides calcined at 400 °C.

Figure 5 and Figure S4 show the catalytic performance of Ce-Co, Mn-Co oxides in the temperature range of 150–375 °C under the traditional heating method. For all reactions, benzene was completely decomposed into H_2_O and CO_2_ without other byproducts of incomplete oxidation via a time-based in situ FTIR with a gas cell. Compared with Ce-Co oxides calcined at 300 and 400 °C, the activity of CeCo-400 is superior to CeCo-300 in general, while the CeCo21-300 is slightly higher than CeCo21-400 in the test range, which is mainly attributed to the enhanced crystallinity of 400 °C calcined samples. In contrast, CeCo12-400 exhibits the highest activity, suggesting that a higher Co ratio for Ce-Co oxides is beneficial to benzene catalytic activity. Compared with CeCo-400 and MnCo-400, the catalytic activity of MnCo-400 is higher than that of the corresponding CeCo-400 samples, which may be attributed to the formation of the spinel structures. The superior activity of spinel structures may be related to their unique atomic-scale cooperativity, where the redox synergy between Mn^3+^/Mn^4+^ and Co^2+^/Co^3+^ at octahedral sites facilitates electron transfer during benzene C-H bond scission [38,43], and three-dimensional oxygen diffusion channels between tetrahedral and octahedral sublattices enable rapid lattice oxygen mobility [44,45]. MnCo11-400 exhibited the highest low-temperature activity and achieved the complete benzene oxidation at 250 °C. For control, tests without a catalyst were conducted under identical conditions. Benzene degradation is negligible (~3.5%) even at 375 °C, confirming that thermal effects alone cannot explain the high conversions achieved with catalysts. What is more, the on-stream reaction stability of CeCo-400 and MnCo-400 was measured at 225 °C for 20 h, and there was no significant decline in the benzene conversion of all the catalysts, indicating high stability of the catalysts (Figure 5b).

### 2.3. Microwave-Enhanced Catalytic Performance of Benzene Oxidation

**Figure 6:**
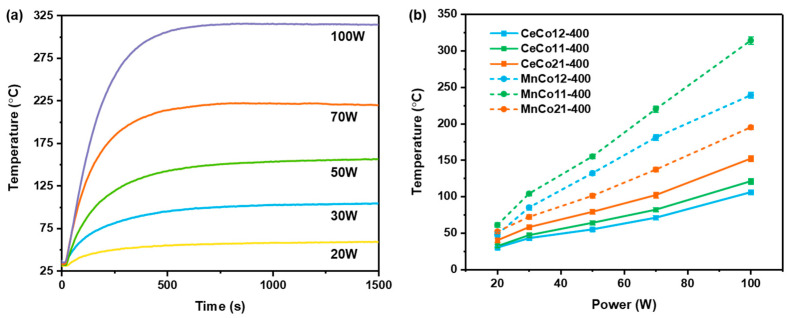
Microwave heating performance, (**a**) heating behavior of MnCo11-400, and (**b**) catalyst temperature at different powers.

Microwave irradiation is effective for the rapid temperature rise of the catalysts. Figure 6a shows temperature–time profiles of MnCo11-400, for example, while other samples show a similar heating behavior. The heating behavior at each power showed a constant and regular temperature rising. The temperature of MnCo11-400 reached a steady state after nearly 10 min microwave irradiation, and the steady-state temperature increased with the power increasing. Figure 6b shows the temperature–power profiles of the CeCo-400 and MnCo-400 samples. The catalyst temperature can be easily controlled according to the microwave output for catalytic reactions under different conditions. MnCo-400 shows a higher temperature than CeCo-400 via microwave irradiation of the same power, suggesting that the microwave heating behavior can be enhanced by adjusting the elemental composition and atomic ratio. The temperature disparities observed between MnCo-400 and CeCo-400 under identical microwave power originate from divergent dielectric loss properties (Figure 6b). MnCo_2_O_4_ exhibits a high loss tangent, enabling efficient microwave-to-thermal energy conversion via small polaron hopping (Co^2+^↔Co^3+^) and Maxwell–Wagner interfacial polarization at grain boundaries [46,47]. In contrast, the lower tan δ values of constituent phases CoO and CeO_2_ reflect weaker electromagnetic coupling, primarily limited to ionic polarization and oxygen vacancy defect oscillations [48,49].

**Figure 7:**
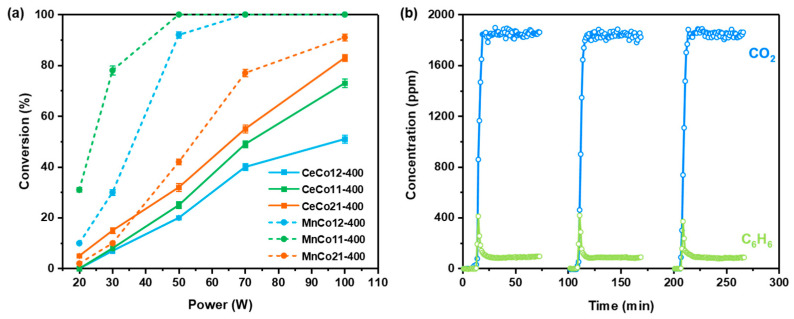
(**a**) Microwave-assisted benzene oxidation performance at different powers and (**b**) stability of benzene oxidation reaction on MnCo11-400 at 30 W for 3 h.

Figure 7 shows the catalyst activities under microwave heating. Since the catalysts can be heated well through microwaves, especially Mn-Co oxides, microwave-assisted benzene oxidation shows high activity. What is more, our previous works also demonstrated that microwave irradiation can facilitate lattice oxygen activity, which is important for benzene oxidation on Mn-based oxides via the MvK mechanism [21,22]. At each output power, benzene was completely transformed into CO_2_ and H_2_O without the generation of other by-products, and the benzene conversion increased with the output power rise for all the catalysts. Energy is efficiently converted into heat under microwave irradiation, providing the necessary energy for the reaction and enabling complete benzene conversion at relatively low power levels (20–100 W). The Mn-Co oxides exhibited higher activity than Ce-Co oxides, as shown in Figure 6a. Among them, MnCo11-400 shows the optimal catalytic performance with the benzene conversion of 78% at 30 W, and 100% at 50 W, which is attributed to the high activity and heating performance. Figure 6b demonstrates the stability of MnCo11-400 over three microwave power on-off cycles, with each test lasting about 1 h. The conversion remained stable at approximately 80% under 30 W microwave irradiation, indicating high stability and insensitivity to microwave intermittent operations of the catalysts.

**Figure 8:**
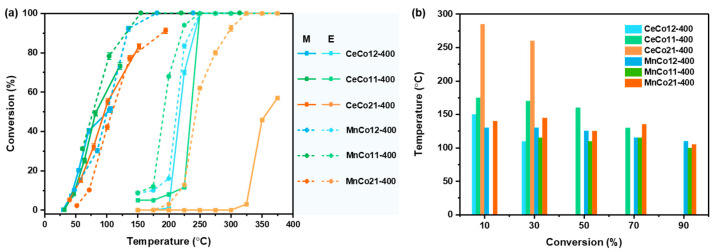
Catalytic performance comparison of 400 °C-calcined Ce-Co and Mn-Co oxides under microwave heating (M) versus conventional electric heating (E): (**a**) Conversion profiles as a function of temperature; (**b**) Temperature differentials (ΔT = T_E_ − T_M_) at fixed benzene conversion levels.

Figure 8 compares the catalytic activity of benzene oxidation on Ce-Co, Mn-Co oxides under microwave and conventional heating modes. Figure 8b plots the reaction temperature difference (ΔT = T_E_ − T_M_) at benzene conversions of 10, 30, 50, 70, and 90% for all catalysts. The electric heating temperatures (T_E_) were recorded via embedded K-type thermocouples in the catalyst bed, while microwave heating temperatures (T_M_) were measured via infrared pyrometry calibrated against thermocouples under reaction gas flow. The temperature required for the reactions on the catalysts is quite lower under microwave heating for the same conversion, where the ∆T is over 100 °C between them, and the highest ∆T is even over 250 °C for CeCo11-400. The selective heating nature of microwaves enables efficient energy absorption at defect sites within metal oxide catalysts, creating localized hotspots [11,12,13]. This induces a non-equilibrium thermal state at which the catalyst surface temperature substantially exceeds the bulk temperature. While traditional thermal catalysis necessitates uniform bulk heating to reach reaction temperatures at catalytic sites, microwave catalysis achieves targeted energy deposition at active sites. This facilitates high-efficiency reactions under markedly reduced thermal conditions, drastically cutting energy consumption.

**Figure 9:**
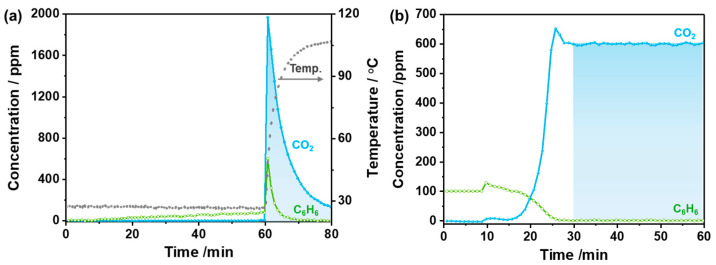
(**a**) Adsorption–desorption and catalytic oxidation on MnCo11-400 (pre-adsorption of 100 ppm benzene in O_2_/N_2_ flow gas at 30 °C for 60 min; microwave power: 30 W) and (**b**) benzene oxidation under conventional electric heating at 200 °C. Catalyst weight: 100 mg; 100 ppm benzene -N_2_ balance: 20% O_2_; gas flow rate: 100 mL·min^−1^.

Taking advantage of rapid microwave heating and the high adsorption performance of the catalysts, a combination of adsorption and microwave-assisted oxidation processes was investigated for benzene removal with low concentrations, which was energy coasting using the traditional thermal oxidation method. As shown in Figure 9a, 100 ppm benzene was pre-adsorbed on MnCo11-400 for 60 min and then subjected to microwave irradiation at 30 W with a benzene-free O_2_/N_2_ stream. The temperature of the catalyst increased rapidly after microwave irradiation, resulting in the formation of CO_2_ with a small amount of benzene spilling. The total amount of benzene decomposition was 1.15 mmol, consistent with the amount of CO_2_ generation (6.8 mmol) for 20 min. Thus, in the combined process, low-concentration benzene was pre-adsorbed onto the catalyst at room temperature and then desorbed from the catalyst surface in a short time and completely oxidized to CO_2_ via microwave irradiation. For comparison, conventional electric heating at 200 °C for 30 min achieves benzene decomposition of 1.35 mmol, with CO_2_ yield stoichiometrically consistent at 8.1 mmol (Figure 9b). Under equivalent reaction conditions, microwave–catalytic treatment via three intermittent 20-min cycles (total 60 min) attains comparable benzene decomposition to 75 min of continuous electric heating. It demonstrates that low-concentration benzene streams can be processed more efficiently through microwave irradiation, establishing an energy-saving approach for enhanced decomposition efficiency.

## 3. Materials and Methods

### 3.1. Catalyst Preparation

CeCo-MOF and MnCo-MOF precursors were prepared using the hydrothermal method. Specifically, for the mole ratio of 1:1, 1.5 mmol of Ce(NO_3_)_3_·6H_2_O (or Mn(NO_3_)_2_·6H_2_O) and 1.5 mmol of (CH_3_COO)_2_Co·4H_2_O were dissolved with 40 mL DMF, and then 0.67g of 1,4-dicarboxybenzene was dissolved in 40 mL DMF to form uniform solutions under stirring for 30 min at first, respectively. All the reagents are purchased from Aladdin in Shanghai, China. The above solutions were mixed evenly and transferred to a 100 mL Teflon-lined autoclave and reacted at 80 °C for 24 h. Then, followed by filtering and washing with DMF and ethanol three times, respectively, the obtained powders were dried at 60 °C for 24 h. Ce-Co, Mn-Co oxides were further obtained with the CeCo-MOF and MnCo-MOF calcination at 400 °C for 3 h, denoted as CeCo11-400 and MnCo11-400. For catalysts with other metal ratios (1:2 and 2:1), the synthesis follows the same method, and the naming convention also applies, with the total metal ion amount fixed at 3 mmol.

### 3.2. Characterization

The crystal phases of the samples were detected via the X-ray diffraction (XRD, Bruker D8 Advance, Berlin, Germany) with Cu Kα radiation (λ = 0.1541 nm) operated at 40 kV and 40 mA. A particle size analysis was performed using Scherrer’s equation (1) applied to the dominant diffraction peaks of each crystalline phase. The surface morphology of the samples was observed via Verios 5 XHR with scanning electron microscopy (SEM, Thermo Fisher Scientific Inc., Waltham, MA, USA). The surface elemental states and absorbed species were characterized through X-ray photoelectron spectroscopy (XPS, Thermo Kalpha, Waltham, MA, USA). N_2_ adsorption isotherm was measured using Micromeritics ASAP 2460 (Micromeritics, Norcross, GA, USA) at 77 K after degassing at 473 K for 2 h. The specific surface area (S_BET_) of the samples was estimated via the Brunauer–Emmett–Teller (BET) model.

(1) $$D=\frac{K\lambda }{\beta \cos\theta }$$

where the following applies: *D*, crystallite size (nm); *K*, shape factor; *λ*: Cu-Kα radiation; *β*: corrected FWHM (radians); and *θ*, Bragg angle.

### 3.3. Catalytic Reactions

**Figure 10:**
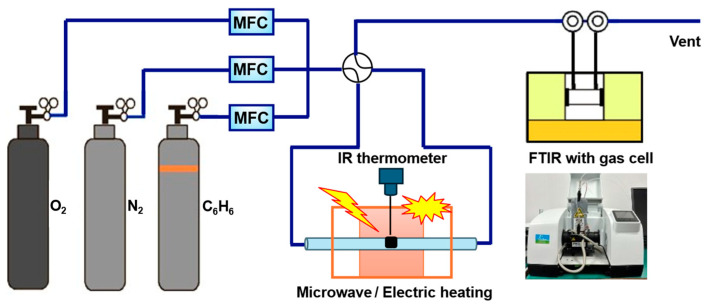
The image of reaction apparatus.

For the case of the catalytic reaction under microwave heating, a 100 mg sample was set up in the quartz tube reactor located in the microwave generation equipment (Figure 10). The catalyst temperature was controlled by regulating the microwave power and measured using an IR thermometer. Before the catalytic reaction, the pretreatment of samples under microwave heating with a power range of 20–100 W was carried out in 20% N_2_ -O_2_ flow gas, and then the heating behavior of the samples was measured at different powers. The catalytic oxidation of benzene was carried out over the catalyst in a fixed-bed flow reactor. The reaction conditions were as follows: 400 ppm benzene–N_2_ balance, 20% O_2_; gas flow rate, 100 mL·min^−1^; and weight hourly space velocity (WHSV), 60,000 mL·g^−1^·h^−1^. The evolution of reactants was recorded through in situ Fourier transform infrared spectroscopy. The reaction under conventional heating was carried out using the same reactor placed in an outer heating cylindrical furnace with the same conditions as microwave heating.

The benzene conversion was calculated according to the following equations:

(2) $$Benzene\;Conversion,\;X\;=\frac{{\left(C_{6}H_{6}\right)}_{in}-{\left(C_{6}H_{6}\right)}_{out}}{{\left(C_{6}H_{6}\right)}_{in}}\times 100\%$$

## 4. Conclusions

This work demonstrates that MOF-derived Mn-Co spinel oxides exhibit exceptional microwave–catalytic performance for benzene oxidation, attributable to synergistically engineered dielectric properties and spinel-facilitated oxygen mobility enhancement. The key findings reveal the following. (1) Low-temperature efficiency: Complete benzene conversion is achieved at a low microwave power of 50 W on MnCo11-400. Microwave-specific localized hotspots significantly lower the reaction temperature of 100–250 °C compared to conventional heating, reducing energy consumption. (2) Structural stability: MnCo11-400 maintains more than 80% conversion over repeated power cycling (3 × 1 h on/off), confirming robustness under intermittent operation. (3) Process innovation: An adsorption-rapid microwave oxidation synergy strategy mineralizes pre-adsorbed low-concentration benzene (1.15 mmol) within 20 min at 30 W, achieving parity with 25 min continuous thermal treatment while cutting energy input by >20%. The tailored Mn-Co spinel structure, with a high defect density and mesoporosity (pore volume: 0.20 cm^3^·g^−1^), synergizes microwave absorption and catalytic function. This work establishes MOF-derived spinel oxides as high-efficiency microwave catalysts for sustainable VOC abatement, with direct implications for low-energy emission control technologies.

## Figures and Tables

**Table 1:** The ratio of ions based on XPS.

Samples	Ce^3+^/Ce^4+^	Mn^3+^/Mn^4+^	O_ads_/O_latt_	^a^ O_vac_%
CeCo12-400	0.15	-	0.26	20.0
CeCo11-400	0.22	-	0.07	21.7
CeCo21-400	0.20	-	0.05	21.1
MnCo12-400	-	1.17	0.20	28.5
MnCo11-400	-	1.20	0.87	33.2
MnCo21-400	-	0.91	0.19	24.4

^a^ The concentration of oxygen vacancies (O_vac_) evaluated through the following equation: O_vac_% = $\frac{A_{O_{vac}}}{A_{O_{latt}+A_{O_{vac}}+A_{O_{ads}}}}$ × 100%.

**Table 2:** Textural properties of Ce-Co and Mn-Co oxides.

Samples	S_BET_/m^2^·g^−1^	Pore Size Distribution/nm	Pore Volume/cm^3^·g^−1^
CeCo12-400	72	21	0.10
CeCo11-400	95	7	0.07
CeCo21-400	102	7	0.05
MnCo12-400	81	23	0.27
MnCo11-400	65	20	0.20
MnCo21-400	62	21	0.19

## Data Availability

The raw data supporting the conclusions of this article will be made available by the authors upon request.

## Supplementary Materials

The following supporting information can be downloaded at: https://www.mdpi.com/article/10.3390/molecules30163388/s1, Figure S1: XRD patterns of CeCo12-300, CeCo11-300, and CeCo21-300; Figure S2: XPS survey scan spectrum of (a) CeCo-400, (b) MnCo-400; Figure S3: SEM images of (a) CeCo12-300, (b) CeCo11-300, (c) CeCo21-300; Figure S4: Catalytic activity of CoMn-300; Table S1: The crystallite size of the catalysts.
