# Supplementary material for: Microwave-Enhanced Catalytic Performance of Benzene Oxidation on MOF-Derived Mn/Ce-Co Oxides
Source: Molecules. 2025 Aug 15;30(16):3388. doi: 10.3390/molecules30163388 (PMC12388452; doi:10.3390/molecules30163388)
Supplement: Supplementary file 1 [file molecules-30-03388-s001.zip › molecules-3796821-supplementary.pdf]

Supporting Information

Microwaved-enhanced catalytic performance of benzene oxidation on MOF-derived Mn/Ce-Co oxides

Shefeng Li[1,2,3], Pengyi Zhao[1], Ziyang Liu[1,2], Chang Wang[1,2], Linling Wang[4], Siyu Ding[1,2,*]

[1] School of Chemical and Environmental Engineering, Wuhan Polytechnic University, Wuhan 430023, China
[2] Pilot Plant of Eco-Environment Chemical Industry and Carbon-Neutral Transformative Technologies, Wuhan 430023, China
[3] Hubei Provincial Engineering Research Center of Soil and Groundwater Pollution Prevention and Control, Wuhan 430023, China
[4] School of Environmental Science and Engineering, Huazhong University of Science and Technology, Wuhan 430074, China
* Correspondence: siyu_ding@hotmail.com

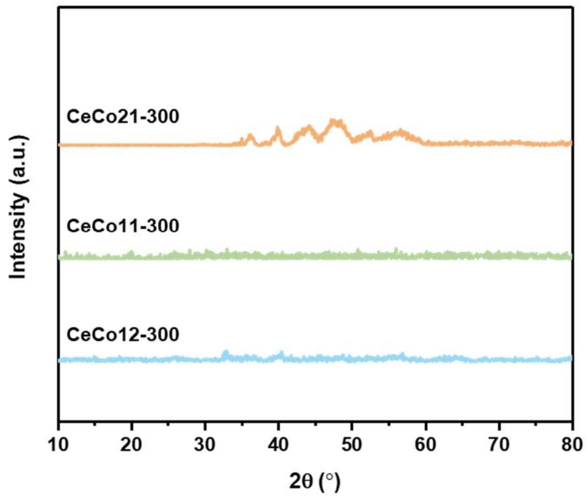

Figure S1. XRD patterns of CeCo12-300, CeCo11-300, and CeCo21-300.

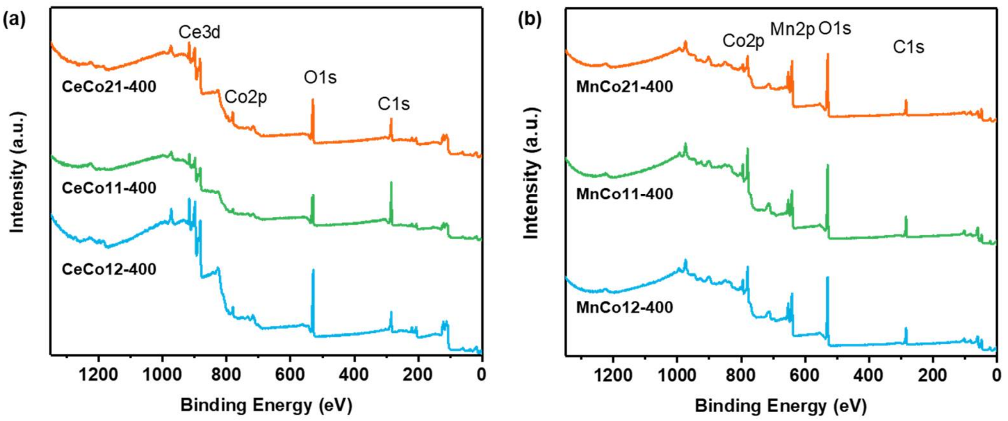

Figure S2. XPS survey scan spectrum of (a) CeCo-400, (b) MnCo-400.

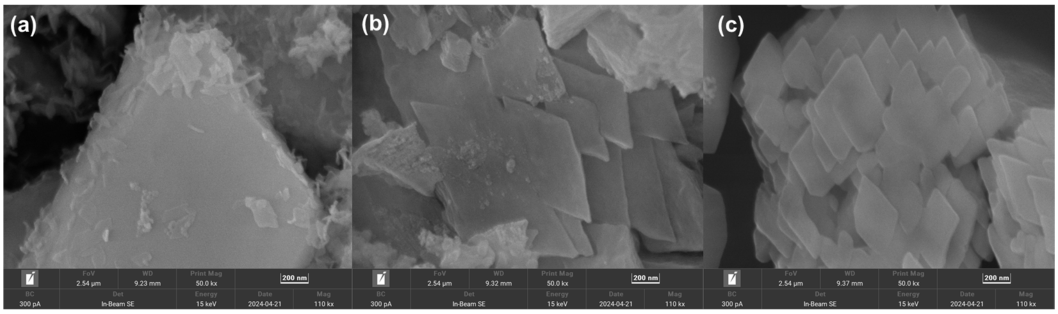

Figure S3. SEM images of (a) CeCo12-300, (b) CeCo11-300, (c) CeCo21-300.

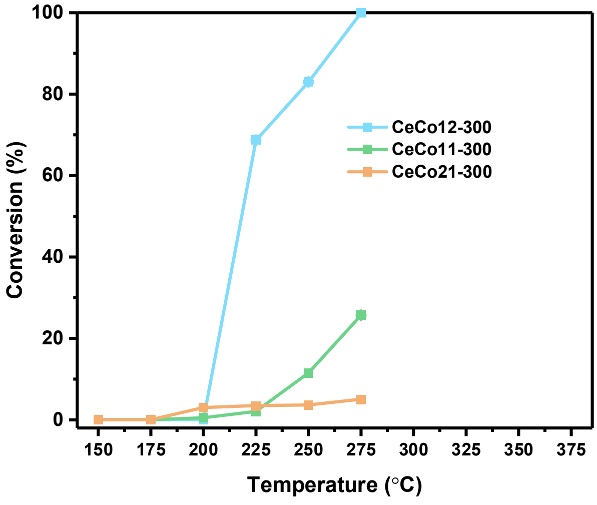

Figure S4. Catalytic activity of CoMn-300.

Table S1. The crystallite size of the catalysts.

Sample	Ratio (Ce/Co, Mn/Co)	Phase	Dominant Peak	Crystallite Size (nm)
CeCo12-400	0.54	CeO_2	(111)	8.1
		Co_3O_4	(311)	11.8
CeCo11-400	1.11	CeO_2	(111)	7.9
		CoO	(111)	12.1
CeCo21-400	2.10	CeO_2	(111)	8.5
		CoO	(111)	12.5
MnCo12-400	0.51	$MnCo_2O_4$	(311)	17.9
MnCo11-400	0.98	$MnCo_2O_4$	(311)	18.2
MnCo21-400	2.03	$CoMn_2O_4$	(311)	15.6
